# Reviewing and renewing the use of beneficial root and soil bacteria for plant growth and sustainability in nutrient-poor, arid soils

**DOI:** 10.3389/fpls.2023.1147535

**Published:** 2023-04-06

**Authors:** Noor Khan, Ethan A. Humm, Akshaya Jayakarunakaran, Ann M. Hirsch

**Affiliations:** ^1^ Department of Molecular, Cell, and Developmental Biology, University of California, Los Angeles, Los Angeles, CA, United States; ^2^ Molecular Biology Institute, University of California, Los Angeles, Los Angeles, CA, United States

**Keywords:** plant growth promoting bacteria, sustainable ecosystems, soil bacteria, endophytes, climate change

## Abstract

A rapidly increasing human population coupled with climate change and several decades of over-reliance on synthetic fertilizers has led to two pressing global challenges: food insecurity and land degradation. Therefore, it is crucial that practices enabling both soil and plant health as well as sustainability be even more actively pursued. Sustainability and soil fertility encompass practices such as improving plant productivity in poor and arid soils, maintaining soil health, and minimizing harmful impacts on ecosystems brought about by poor soil management, including run-off of agricultural chemicals and other contaminants into waterways. Plant growth promoting bacteria (PGPB) can improve food production in numerous ways: by facilitating resource acquisition of macro- and micronutrients (especially N and P), modulating phytohormone levels, antagonizing pathogenic agents and maintaining soil fertility. The PGPB comprise different functional and taxonomic groups of bacteria belonging to multiple phyla, including *Proteobacteria, Firmicutes, Bacteroidetes*, and *Actinobacteria*, among others. This review summarizes many of the mechanisms and methods these beneficial soil bacteria use to promote plant health and asks whether they can be further developed into effective, potentially commercially available plant stimulants that substantially reduce or replace various harmful practices involved in food production and ecosystem stability. Our goal is to describe the various mechanisms involved in beneficial plant-microbe interactions and how they can help us attain sustainability.

## Introduction

The world’s population increased to approximately 8 billion people in 2022. This increase in the human population will further imperil ecosystems and soils already stressed by climate change and anthropogenic activities, severely threatening food production particularly in arid and semi-arid regions. Climate change has intensified the various stressors that negatively affect plant growth all over the globe. For the next few decades, we will need to overcome significant challenges to feed all the world’s people and their agricultural animals, as well as to maintain stability for natural environments. This is a problem that will only intensify with time (Sprent and Sprent, 1990). Hence, there is a great need for abiotic stress-tolerant and sustainable solutions that allow for food production and maintenance of natural sites despite an increasing world population, but without the current excessive use of non-biological inputs that exacerbate soil degradation. Plant growth-promoting bacteria (PGPB) function and promote plant growth even in unfavorable, non-conducive soil conditions (Bashan and Holguin, 1998; Hartmann et al., 2008; Morales-Cedeño et al., 2021), and can restore degraded soils. In this review, we highlight the beneficial bacteria residing in the soil and root regions of plants that are potential solutions to the pressing problems. We analyze the recent literature regarding the utilization of beneficial bacteria and their products to enhance plant growth and for protection against phytopathogens.

## Beneficial rhizobiome and nodule microbiome interactions

The German agronomist and plant physiologist, Lorenz Hiltner, who coined the term “rhizosphere” in 1904, described it as the area around the plant root that is inhabited by a unique population of organisms, including nematodes, fungi, bacteria, and arthropods. This dynamic ecosystem, the rhizobiome, contains numerous deposits including microbial and plant-derived secretions, many of which, such as mucilage, are passively released from plant tissues due to osmotic differences between the soil solution and the cortical cells of the plant roots (Hiltner, 1904; Hartmann et al., 2008). Enriched by these root exudates, the rhizosphere serves as a hotspot for microbes, particularly beneficial bacteria and fungi that positively affect plant growth and health (Reinhold-Hurek et al., 2015). Jamil et al. (2022) highlighted the dynamic importance of rhizospheric plant-microbe interactions that benefit plant productivity. Their review and many other studies referenced therein emphasize the inter- or intraspecies communication that occurs *via* the quorum sensing (QS) mechanism, which involves cell density-dependent coordination. Cell density is detected by an accumulation of QS molecules or autoinducers. Once a threshold concentration of QS molecules is reached, bacteria display coordinated behaviors including mating, release of antimicrobial compounds, biofilm formation, antibiotic resistance, virulence, and light production, among other activities. The best studied QS molecules are acyl homoserine lactones (AHLs) produced by Gram-negative bacteria. In addition to AHLs, a wide variety of QS molecules has been identified, including peptides produced by Gram-positive bacteria, and autoinducer-2 (AI-2), which is produced and recognized by both Gram-negative and Gram-positive bacteria (Li and Nair, 2012). PGPB can interfere with quorum sensing and pathogenesis by degrading QS molecules produced by phytopathogens. For example, *Pseudomonas segetis* strain P6 has been shown to interfere with quorum sensing, degrading a wide range of AHLs produced by phytopathogens and reducing disease severity on potato, carrot, and tomato (Rodríguez et al., 2020). While the role of quorum sensing in orchestrating the production of virulence factors by phytopathogens has been well established, beneficial bacteria also utilize quorum sensing to coordinate activities related to plant growth promotion. Xiong et al. (2020) demonstrated AI-2 regulates motility, biofilm formation, and root colonization by *Bacillus velezensis* SQR9. Similar results are seen in Gram-negative bacteria; a mutant of *Paraburkholderia phytofirmans* PsJN that is impaired in AHL production shows reduced growth promotion and colonization of *Arabidopsis thaliana* roots (Zúñiga et al., 2013).

Although the rhizobiome contains both beneficial and non-beneficial microbes, a healthy balance results in a selective build-up of a community of favorable microorganisms, which not only protects a healthy plant from pathogens, but also enables colonization by beneficial microbes that perform various plant growth functions such as siderophore production, phytohormone synthesis, and phosphate (P) solubilization. P-solubilizing bacteria have attracted significant attention because of their agronomic use as a safe P-fertilizer in place of costly commercial P-chemical fertilizers (Oteino et al., 2015). Microbes that solubilize inorganic phosphate do so primarily by secreting organic acids whereas organic phosphate is mineralized by the action of various enzymes, including phosphatases and phytases (Alori et al., 2017). Indeed, Cook et al. (1995) postulated that plants modulate their rhizosphere microbiomes to ensure their own benefit by selectively stimulating microorganisms to synthesize products that are beneficial to plant growth and health. Studies conducted by Li Z et al. (2021) demonstrated that the legume *Astragalus mongholicus*, when challenged by the root-rot pathogen *Fusarium oxysporum*, recruited beneficial bacteria such as *Stenotrophomonas, Achromobacter, Pseudomonas*, and *Flavobacterium* to the rhizospheric region to act as biocontrol agents. Zia et al. (2021) further demonstrated that rhizosphere management could lead to increased nutrient efficiency in the soil to boost both plant growth and productivity.

Plant-microbe interaction research has been focused on three major symbioses: 1) the mycorrhizal symbiosis between plants and fungi (Bonfante and Genre, 2010; Begum et al., 2019); 2) biological nitrogen fixation and other positive interactions between plants and bacteria (Hirsch and Fujishige, 2012; Kucho et al., 2017; Bellabarba et al., 2019); and 3) pathogenesis (Dodds and Rathjen, 2010; Kachroo and Robin, 2013; Wirthmueller et al., 2013; Pollak and Cordero, 2020; Li J et al., 2021). Many of these systems are well-characterized and provide insights into common and diverged trans-kingdom signaling systems operative in plant-microbe communication. The most intriguing among these is nitrogen fixation. It is an irony of the botanical world that despite 80% of Earth’s atmosphere being composed of dinitrogen gas, it cannot be used by plants as such and must be ‘fixed’ by various bacteria. For legumes, several different genera of the Alphaproteobacteria (including *Rhizobium, Bradyrhizobium, Mesorhizobium*, and *Ensifer*) and Betaproteobacteria (*Paraburkholderia, Cupriavidus*) form root nodules and perform nitrogen fixation, whereas in association with non-legumes, a filamentous actinomycete in the genus *Frankia* is responsible (Hibbs and Cromack, 1990). Additionally, many studies of nitrogen-fixing properties among the Gram-positive Actinobacteria revealed that some species of *Arthrobacter, Agromyces, Corynebacterium, Micromonospora*, and *Streptomyces* have nitrogen-fixing capacity (Sellstedt and Richau, 2013; Svistoonoff et al., 2014).

Bacterial inoculants also modulate the plant microbiota and influence microbial ecological interactions. Sun et al. (2022) demonstrated that a well-established biocontrol bacterium, *B. velezensis* SQR9, alters rhizosphere microbiomes by changing the abundance of other beneficial bacteria. The recruited strains can serve as cooperative partners of strain SQR9 and assist in plant growth promotion. The rhizosphere microbiome adapts the plant to harsh conditions, including aridity, contamination from heavy metals or organic molecules, as well as nutrient deficiency.

Compant et al. (2019) reviewed the importance and functionalities of the bacterial-plant microbiome and discussed challenges and concepts regarding tailored selection of desired microbes for microbiome improvement employing appropriate agro-management practices. The rhizobiome is the most well-studied because it offers a plentiful source of PGP microorganisms investigated by many researchers. Although the internal tissues of roots or root nodules are generally not considered in an inventory of PGPB, many bacteria demonstrate a different degree of selection beyond that of surface interactions. Bacteria enter the developing root nodule either by “crack entry”, which involves intercellular penetration, or *via* infection threads. Legume root nodules thus become another important reservoir for microbial endophytes and can augment rhizobiome functions (Martinez-Hidalgo and Hirsch, 2017). Legumes develop root nodules, which are elicited by the cooperative interaction of a legume with its cognate rhizobium. These underground, carbon-storing plant organs are thus important reservoirs for microbial endophytes, some of which may augment rhizobiome functions. For example, the endophytes uncovered in the alfalfa nodule microbiome include species of *Rhizobium*, *Ensifer*, *Pseudomonas*, *Pantoea*, *Nocardioides*, *Bosea*, *Variovorax*, as well as several fungal species (Hansen et al., 2020; Ilyas et al., 2022). *Variovorax* (“variable feeder”) species isolated from alfalfa root nodules exhibit numerous plant growth promoting activities including synthesis of siderophores, solubilization of phosphate, induction of stress tolerance, phytohormone production, and biocontrol. Two *Variovorax* species (*V. paradoxus* and *V. boronicumulans*), isolated from alfalfa nodules (E. Humm, A. Jayakarunakaran, et al., unpubl. results), were found to contribute to plant growth promotion *via* numerous microbial pathways, such as degrading industrial compounds such as herbicides, pesticides (Zhang et al., 2012; Brandt et al., 2014), acrylamides (Liu et al., 2015) and neonicotinoids (Zhang et al., 2012).

## Biofilms and exopolysaccharide production

Irrespective of the mechanism of action employed by beneficial bacteria, the first step towards benefiting the plant is the direct interaction between microbes and plant roots, followed by the effective colonization of plant roots by bacteria. Chemotaxis and motility are active mechanisms that play a major role in this process (Colin et al., 2021). de Weert et al. (2002) demonstrated that chemotaxis of *Pseudomonas fluorescens* WCS365 towards malic and citric acids in tomato root exudate was the major factor in competitive tomato root colonization. In the natural environment, bacteria usually exist in multicellular aggregates or biofilms, in which the cells are embedded in a matrix of extracellular polymeric compounds attached to a surface. Living in biofilms helps protect bacteria from non-conducive environmental conditions, and biofilm formation appears to be an important factor in the disease cycle of bacterial pathogens for both animals and plants (Parsek and Fuqua, 2004). Root-associated pseudomonads and bacilli have been studied extensively in this regard. Espinosa-Urgel et al. (2002) showed that *Pseudomonas putida* responds rapidly to the presence of root exudates in soils, which results in bacterial aggregation at various root colonization sites and in doing so, establishes stable biofilms. *Azospirillum brasilense* and related species are also motile and form biofilms (Jijón-Moreno et al., 2019). Bagheri et al. (2022) described the biological and beneficial effects of when two bacteria, *Azospirillum oryzae* NBT506, a nitrogen-fixing species, and *Bacillus velezensis* UTB96, a PGPB, were grown in co-culture. The co-culture system showed that a more stable biofilm formed and that indole acetic acid (IAA) production, was enhanced in comparison to the monocultures. Studies involving direct observations of bacteria adherent to plant surfaces have also revealed multicellular assemblies variably described as microcolonies, aggregates, and cell clusters. In addition, seed-associated bacteria are another source of beneficial endophytes and may colonize the incipient rhizosphere *via* chemotaxis and motility.

Although rhizobiomes and seed microbiomes are not the only sources of beneficial bacteria for plant growth, they are the most studied. In contrast, internal tissues are often not subjects for study although they are also a source of endophytes.

## Biostimulants from hotter, drier soils

With changing climatic conditions and Earth becoming warmer, there is a critical need for beneficial bacteria that survive and function in fluctuating environments. PGPB isolated from plants or soils in hot and dry climatic zones are predicted to adapt to changing conditions faster than the plants living in these environments. To test this hypothesis, Khan et al. (2020) employed cultivation-dependent methods to isolate bacteria from the Negev Desert in Israel. Several bacterial strains were isolated and tested on corn in both greenhouse and small field studies. One unusual abiotic-stress tolerant strain, *Dietzia cinnamea* 55, significantly enhanced the overall plant health of corn in comparison to untreated controls. Previously, Kumar and Gera (2014) reported that *Brevundimonas* sp. MDB4, which was isolated from the rhizospheric soil sample of sugarcane growing in an arid region in India also promoted plant growth. The bacterial isolate was tested and found to be a multi-trait PGPR that could not only fix biological nitrogen but also significantly enhance the growth of Bt cotton (var. RCH 134). Many studies credit the ability of bacteria supporting plant growth in stressed environments to their capability of reducing levels of “stress ethylene” by producing the enzyme 1-aminocyclopropane-1-carboxylate deaminase (ACCD) (Gupta and Pandey, 2019). A recent review by Singh et al. (2022) details the role of ACC deaminase in breaking down ACC (an immediate precursor of ethylene) to ammonia and α-ketobutyrate, thereby reducing the level of ethylene inside the plants and improving their resistance to various stresses such as salinity, drought, flood, and various pathogens. Zarei et al. (2020) reported that a combination of four *Pseudomonas fluorescens* strains (P1, P3, P8 and P14) improved the root growth of sweet corn in comparison to the uninoculated controls through the synergistic effects of the different strains in the production of ACC deaminase, auxin synthesis, mineral phosphate solubilization, and the production of siderophores. This combination also significantly improved the yield traits of sweet corn at different levels of irrigation by reducing stress ethylene and increasing water absorption and nutrients. Various consortia of ACC deaminase-producing bacteria have also been shown to promote the growth of French beans (*Phaseolus vulgaris*) and holy basil (*Ocimum sanctum*) under salinity and cold stress, respectively (Gupta and Pandey, 2019; Singh et al., 2020), as well as *Arabidopsis* in soils contaminated with cadmium and lead (Grobelak et al., 2018).

Climate change is also expected to exacerbate soil salinization through various mechanisms including increased seawater intrusion and reduced leaching of salts from soil due to perturbed precipitation patterns and increased evaporation. These deleterious effects are more pronounced in semi-arid and arid regions (Hassani et al., 2021). However, halophytic plants and their associated microbiomes can provide insight into how crop growth can be improved in saline soils. For example, Yuan et al. (2016) investigated the microbiome of the halophyte *Suaeda salsa*, revealing that the rhizosphere and internal root tissues of *S. salsa* are enriched with microbes encoding genes related to salt stress tolerance. Additionally, salt-tolerant bacteria isolated from the rhizosphere of halophytes have been shown to improve salinity stress in agricultural crops such as alfalfa (Kearl et al., 2019), wheat (Sorty et al., 2016), and maize (Ullah and Bano, 2015).

## PGPB as heavy metal accumulators and biocontrol agents

The release of heavy metals into the environment by mining, construction, fossil fuel production, and a multitude of other industrial activities leads to soil degradation, surface and groundwater contamination, and other assaults on the health of plants and animals, including humans. Various PGPBs have been shown to both tolerate and accumulate environmental heavy metals. Plant beneficial bacteria including *Rhizobium, Ensifer, Pseudomonas, Bacillus*, and *Paraburkholderia* enhance the performance of plants used for phytoremediation of heavy metals and organic pollutants, including *Lathyrus sativus* (Abdelkrim et al., 2019) and *Populus* (Taghavi et al., 2005). Choudhary et al. (2012) isolated strains of *Bacillus, Serratia*, and *Arthrobacter*, often reported to be PGPBs, from uranium mine waste; these bacteria were able to tolerate and accumulate uranium and other heavy metals. Additionally, *Variovorax* spp. are known to bioremediate soils contaminated by heavy metals or organic compounds (Liu et al., 2013; Satola et al., 2013). Benedek et al. (2021) showed the applicability of *Variovorax paradoxus* strain BFB1_13 in the bioremediation of BTEX contaminated sites. Monoaromatic pollutants such as benzene, toluene, ethylbenzene and isomers of xylene are referred to as BTEX compounds. Our unpublished data show that *Variovorax* spp. can tolerate heavy metals such as copper and nickel, and improve plant growth in contaminated soils. Three of the *Variovorax* nodule isolates, *V. paradoxus* SPNA7, *V. boronicumulans* EBFNA2 and *V. paradoxus* 2u118, were tested to tolerate cupric chloride and nickel chloride under *in vitro* conditions. Growth chamber experiments showed an improved vigor index for alfalfa seedlings irrigated with 100 ppm cupric chloride and inoculated with *Variovorax* strains in comparison to the uninoculated controls.

Biological control or biocontrol is the use of bacteria or their molecules in limiting the growth and spread of plant pathogens, including bacteria and fungi, potentially leading to healthier disease-free plants. These antagonistic bacteria are a great hope for reducing the use of pesticides in agricultural soils. Effective biocontrol agents can promote plant growth by directly inhibiting the growth of phytopathogens through a variety of means, including production of antimicrobial compounds and competition for resources. Some beneficial bacteria produce siderophores, high-affinity iron-chelating compounds that provide their producers a competitive advantage in colonizing the plant roots, thereby excluding pathogenic microbes. Different categories of bacterial siderophores, mainly hydroxamates, carboxylates, and catecholates have been identified, and they show varied abilities to sequestrate iron *in vitro* (Wang et al., 2021). PGPB can also outcompete phytopathogens by producing biofilms. For example, Bais et al. (2004) showed that robust biofilm formation by *Bacillus subtilis* is crucial for colonization of *Arabidopsis* roots and protection against infection by *Pseudomonas syringae.* In addition to directly antagonizing pathogens, PGPB elicit systemic resistance responses in plants to prime them against pathogen attack. Perhaps the best studied priming interaction is induced systemic resistance (ISR), whereby root-colonizing PGPB produce molecules and compounds known as elicitors (e.g., microbe associated molecular patterns or MAMPs proteins including flagellin, cell wall fragments as well as volatile organic compounds; and siderophores that sensitize the entire plant to potential pathogen attack, which led to a faster and higher amplitude defense response. This systemic response is mediated primarily by the plant hormones jasmonic acid (JA) and ethylene (ET). Interestingly, PGPB that activate ISR also induce iron deficiency responses in host plants, in part because of the involvement of ET in both ISR and responses to iron deficiency (Romera et al., 2019). Besides the elicitors mentioned above, quorum sensing molecules have also been implicated in systemic resistance responses. For example, Hu et al. (2018) showed that AHLs elicit ISR in tomato to confer resistance to *Botrytis cinerea*, whereas Hernández-Reyes et al. (2014) demonstrated that the induction of systemic resistance in barley, wheat, and tomato by *Sinorhizobiun meliloti* is dependent on production of AHLs.

Of the many phytopathogenic fungi in the soil, a frequent phytopathogen is *Fusarium oxysporum* (Fravel et al., 2003). *F. oxysporum* causes vascular wilt diseases in numerous plant species, causing significant crop loss. Many *Fusarium* species also produce mycotoxins that negatively affect human and livestock health (Khan et al., 2017; Munkvold, 2017). Numerous fungicides are successfully used for crop protection, but they may have side effects on humans. Application of such toxic fungicides requires that the sprayer wear personal protective equipment (PPE) to ensure limited direct exposure. Also, because these chemicals cause skin and eye irritation, low doses of the fungicides are applied, and must be reapplied multiple times during the growing season. Biocontrol methods using plant microbiome bacteria, specifically bacilli and their extracted metabolites to inhibit fungal infestation is much safer for soil health and human microbiome health than consuming plants treated with the current arsenal of fungicides and pesticides, many of which accumulate and persist in the soil and human body as well. Bacilli are preferred because of their spore-forming ability that enables them to persist and tolerate fluctuating environmental conditions. Cook et al. (1995) reported that antibiotics produced by rhizobacteria play a significant role in protecting plants against disease development and infection by phytopathogens. Numerous compounds are produced by plant-associated bacteria, and it is likely that this diversity is targeted towards specific pathogens.

A number of studies have established the use of plant growth-promoting *Bacillus* species, as an effective and environmentally sustainable method to protect crops from phytopathogens and to improve plant health (Fira et al., 2018; Chen et al., 2020; Kim et al., 2021; Li PS et al., 2021). *Bacillus* species synthesize various types of lipopeptides with specific activities against plant pathogens, which gives them a special value in agricultural, biotechnological, and pharmaceutical industries (Villegas-Escobar et al., 2013; Shafi et al., 2017). Lipopeptide antibiotics are antimicrobial, antifungal, antitumor, and have enzyme inhibitory activities that kill fungal pests, but do not negatively affect humans (Medeot et al., 2020). Our studies identified *B. subtilis* 30VD-1 as an effective antagonist against phytopathogenic *F. oxysporum* in both *in vitro* and *in planta* experiments (Khan et al., 2018). We reported the antagonistic efficacy of the crude butanol extract collected from the bacterial culture filtrate (CF) against *F. oxysporum* (**Supplemental Figure 1**). Furthermore, test results with *Caenorhabditis elegans* (a test for virulence) as well as strain 30VD-1’s compatibility with other known PGPBs, particularly species of *Rhizobium, Mesorhizobium, Paenibacillus*, and *Paraburkholderia*, support the non-harmful nature of 30VD-1 and its secreted metabolites. We isolated and identified fengycin (data not shown) from *B. subtilis* 30VD-1. It is a non-volatile component of the metabolome and the most likely to be responsible for biocontrol ability against *F. oxysporum*. Continued studies of biocontrol agents are necessary to develop biosafe fungicides. The use of biological control agents to manage the spread of phytopathogens and to control disease development in plants is being widely explored as a safer alternative to the commonly used agrochemicals. Unintentionally, the excessive use of such chemicals, in part because of government- and industry-funded support, has led to degraded soils, groundwater contamination, and high fertilizer and pesticide prices. As the human population continues to increase, there is a dire need to correct these and other emerging environmental and potential medical problems. This new Green Revolution aims to increase food supply and simultaneously lower environmental impact by developing sustainable agricultural systems *via* biocontrol agents and PGPBs.

## Synthetic microbial communities

Various studies have shown that using a combination of compatible beneficial microbes results in more consistent positive plant response and function in comparison to using single strain inoculants. Bioinoculants based on microbial consortia may include bacteria of different species, or both beneficial bacteria and fungi (Woo and Pepe, 2018; Santoyo et al., 2021). Boonchan et al. (2000) studied the biodegradation of high molecular weight polycyclic aromatic hydrocarbons (PAHs) in liquid media as well as in soil using a defined fungal-bacterial coculture. It was observed that a combination of *Stenotrophomonas maltophilia* VUN10010, and uncharacterized bacterial consortium VUN10009, which includes the fungus *Penicillum janthinellum* VU010201, effectively utilized pyrene as the sole C-source, degrading PAHs, when used as a combination in coculture in contrast to single inoculations. Similarly, Baas et al. (2016) reported a novel consortium called Mammoth P™ comprised of the bacteria *Citrobacter freundii, Enterobacter cloacae, P. putida* and *Comamonas testosteroni.* It was further demonstrated that the use of Mammoth P™ could solubilize phosphate effectively and increase plant emergence, health, and productivity of a variety of plant species including wheat, various herbs, and turf grass. However, the use of any potential pathogen such as *E. cloacae* needs rigorous certification of its biosafety. Martínez-Hidalgo et al. (2019) discussed various methods for evaluating the biosafety aspect of the beneficial bacteria prior to their release into the environment for commercial uses.

**Figure 1:**
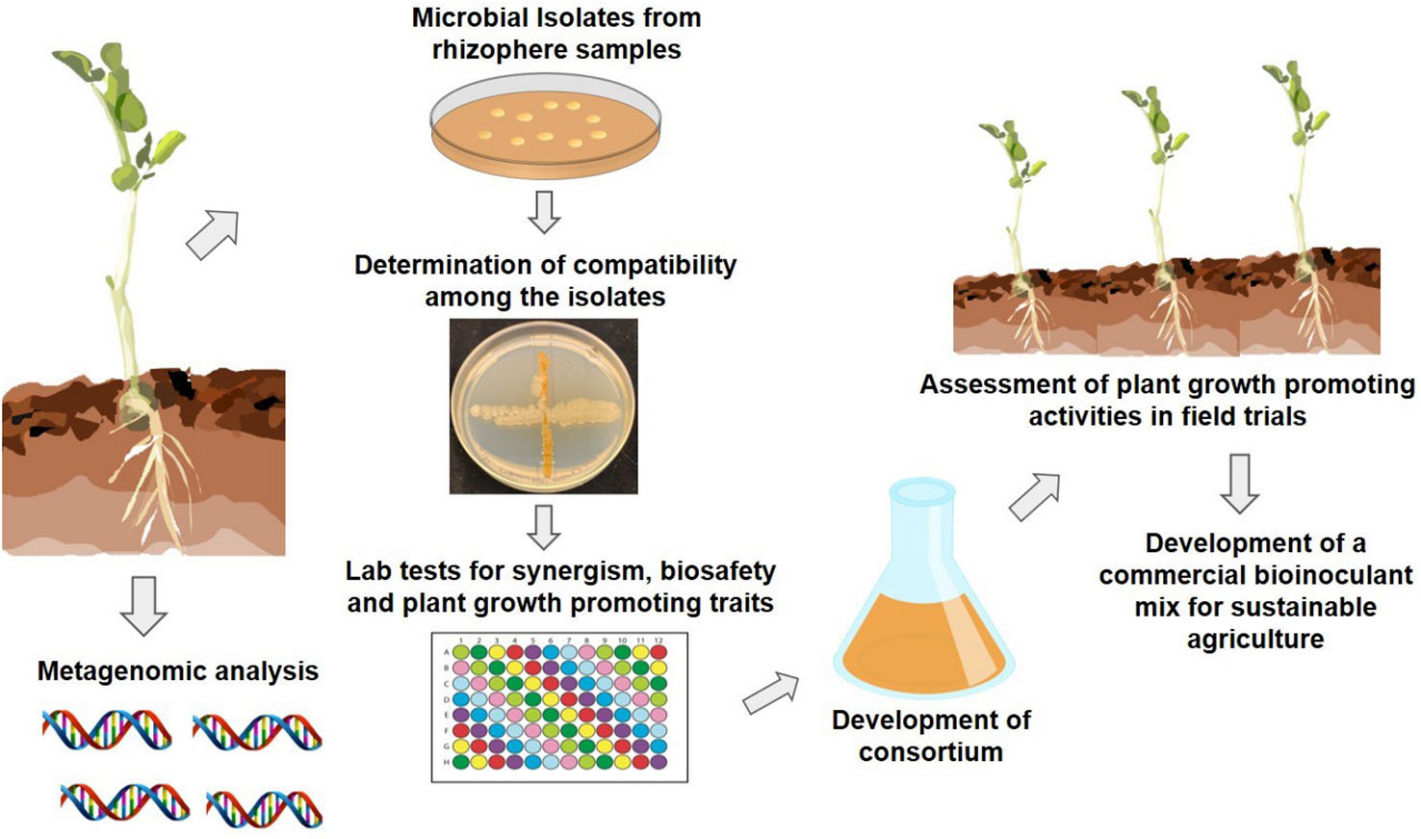
Schematic representation of developing a stable synthetic microbial community for commercial use.

Numerous researchers have shown that the beneficial microbial communities residing in the plant rhizosphere and soil create a functional bond with their hosts and express the beneficial traits capable of enhancing a plant’s performance. What is important is not just the identification of beneficial microbial traits, but their expression under adverse environmental conditions as well. A recently introduced approach of applying ecological concepts and genetics in designing inoculants that are agriculturally relevant is aiding the development of synthetic microbial communities (SynComs). With advances in computational methods, sequencing technologies and analytical tools, our understanding of various facets of plant microbiomes has improved, and it is now known that tailored diverse microbiota can be selected, assembled, and introduced into synthetic communities that are more stable and less complex and can enhance crop resilience to environmental stresses (de Souza et al., 2020). **Figure 1** demonstrates steps involved in developing a synthetic microbial community using bacteria that are rigorously selected, and are more resilient, abiotic-stress tolerant, and stable in comparison to single strain inoculants. Also, it is likely that microbes in SynComs have better chances of survival under changing climatic conditions.

Unlike natural microbial communities, the species in SynComs are known and the community is simple and controllable leading to a better understanding of the mechanisms of interaction between the microbial community and plant (Großkopf and Soyer, 2014). For example, Haruta et al. (2002) established a stable microbial community with high cellulose degradation ability. They used composting materials as the source of microorganisms and constructed a microbial community that degraded more than 60% of rice straw within 4 days at 50°C. The community was further characterized using denaturing gradient gel electrophoresis (DGGE). **Table 1** summarizes some of the recent attempts on developing useful Syncoms that have shown promise *in planta*.

**Table 1:** Examples of synthetic microbial communities (SynComs) and their beneficial effects on crop plants.

SynComs ID and bacterial components	Effect	Reference
Synthetic community of seven strains: *Enterobacter cloacae, Stenotrophomonas maltophilia, Ochrobactrum pituitosum, Herbaspirillum frisingense, Pseudomonas putida, Curtobacterium pusillum*, and *Chryseobacterium indologene*	Inhibited the phytopathogenic fungus *Fusarium verticillioides*, and development of seedling blight disease in maize	Niu et al., 2017
Two SynComs (SynCom 1 comprising of 25 isolates from the rhizosphere of tomato grown in suppressive compost; SynCom 2 comprising of *Bacillus* isolates from the same collection)	Promoted tomato growth and had a protective effect on tomato against *F. oxysporum* f. sp. *lycopersici*.	Tsolakidou et al., 2019
A bacterial SynCom composed of 185 isolates excluding the *Burkholderia* isolates	Increased accumulation of phosphate in *Arabidopsis* shoots under P starvation.	Finkel et al., 2019
Synthetic community of 6 *Pseudomonas* strains from garlic rhizosphere (*P. cedrina, P. baetica, P. migulae, P. fluorescens, P. reinekei* and *P. frederiksbergensis*	SynCom inoculation caused enhancement in plant growth parameters of radish seedlings.	Zhuang et al., 2021
Disease-resistant simplified synthetic community: *Stenotrophomonas* sp., *Rhizobium* sp., *Ochrobactrum* sp. and *Advenella* sp.	Activated induced systemic resistance in *Astragalus mongholicus* against *Fusarium* root rot disease	Li Z et al., 2021
Jizan SynCom C of five bacterial strains (*Massilia* sp. SA087, *Enterobacter* sp. SA187, *Ensifer* sp. SA403, *Bacillus* sp. SA436, and *Streptomyces* sp. SA444) isolated from the root of the desert plant *Indigofera argentea*	Protected tomato plants growing in a non-sterile substrate against a high salt stress.	Schmitz et al., 2022
SynCom of 4 PGP species: *Arthrobacter* sp. MH680887, *Enterobacter* sp. MT158576, *Brevibacterium* sp. MH680885, *Plantibacter* sp. MH680891	SynCom seed dressing can efficiently increase germination rate and early growth parameters of cotton plants.	Kaur et al., 2022
SynCom 1(C1): *Pseudomonas* sp. B5, *Pseudomonas* sp. B11, *Pseudomonas* sp. B12, *Pseudomonas* sp. P25	Of the 10 SynComs developed, SynCom 1 (C1), when compared to the controls, all exhibited stronger antifungal levels and significantly reduced the wheat root rot caused by *Rhizoctonia solani* AG8.	Yin et al., 2022
SynCom 2(C2): *Chryseobacterium* sp. B7, *Chryseobacterium soldanellicola* P38, *Chryseobacterium* sp. P43		
SynCom 3(C3): *Sphingomonas* sp. B17, *Cupriavidus campinensis* B20, *Asticcacaulis* sp. B27, *Rhodococcus erythropolis* B43		
SynCom 4(C4): *Cupriavidus campinensis* B20, *Asticcacaulis* sp. B27, *Rhodococcus erythropolis* B43, *Chryseobacterium soldanellicola* P38		
SynCom 5(C5): *Cupriavidus campinensis* B20, *Rhodococcus erythropolis* B43, *Janthinobacterium lividum* BJ, *Chryseobacterium soldanellicola* P38		
SynCom 6(C6): *Streptomyces* sp. B6, *Chryseobacterium* sp. B7, *Pseudomonas* sp. B12, *Sphingomonas* sp. B17		
SynCom 7(C7): *Pseudomonas* sp. B5, *Streptomyces* sp. B6, *Chryseobacterium* sp. B7, *Pseudomonas* sp. B11		
SynCom 8(C8): *Pseudomonas* sp. B12, *Sphingomonas* sp. B17, *Cupriavidus campinensis* B20, *Asticcacaulis* sp. B27		
SynCom 9(C9): *Pseudomonas* sp. B12, *Rhodococcus erythropolis* B43, *Janthinobacterium lividum* BJ, *Pedobacter* sp. P44		
SynCom 10(C10): All 14 bacterial strains		

## Conclusion and future prospects

**Figure 2:**
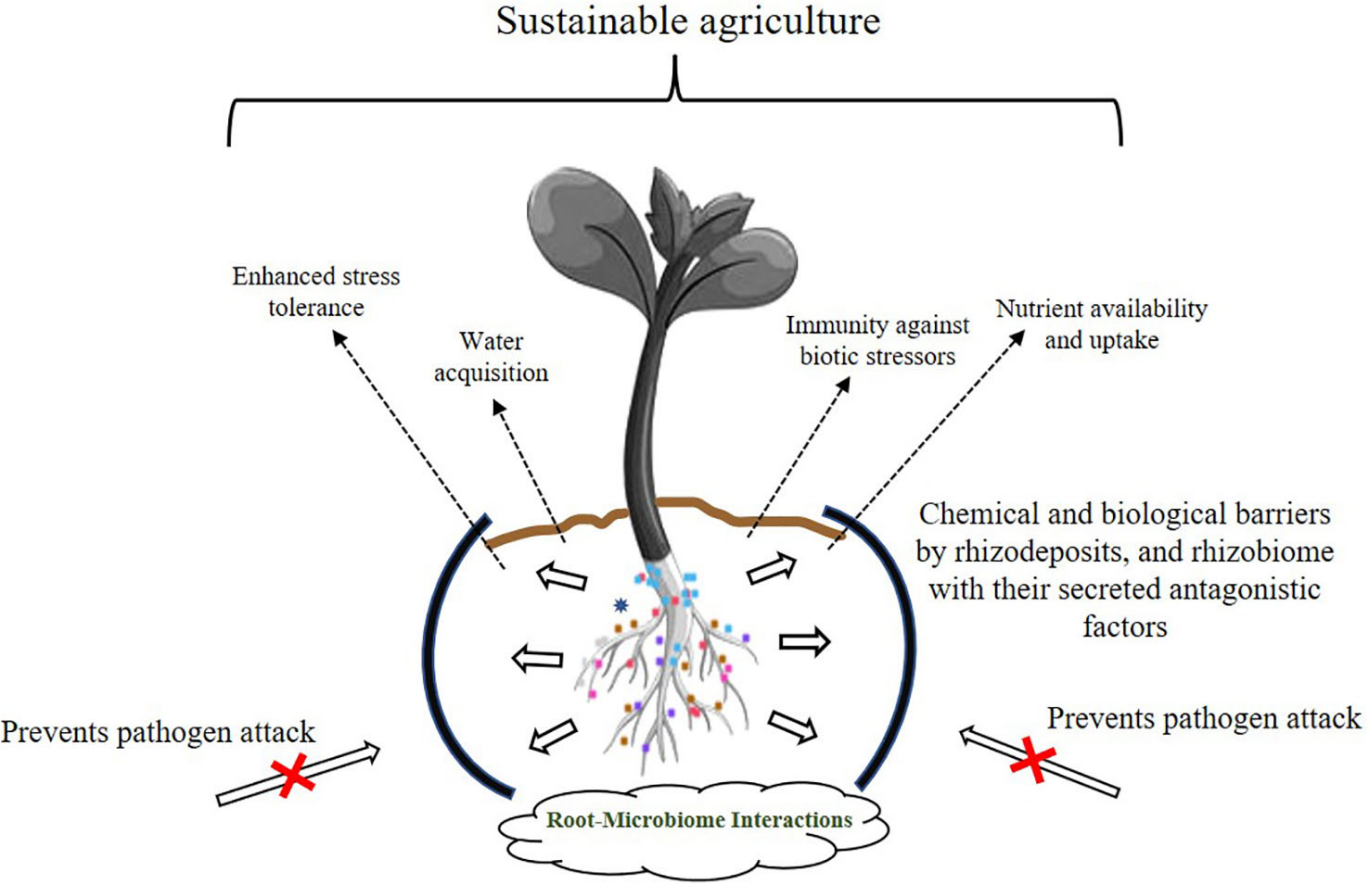
Insights into the tripartite plant-microbe beneficial interactions. Various strategies of plant growth promotion by the rhizobiome are shown. Rhizosphere microorganisms establish beneficial interactions with plants that promote overall plant growth and development. Dashed arrows indicate the benefits provided by the dynamic interaction occurring among rhizosphere microflora-plant-soil microbes.

In natural ecosystems, plants are colonized aboveground and belowground, both externally and internally by mutualistic, commensal, parasitic, and pathogenic organisms. It is this plant-microbe interaction that makes the soil alive (Nadeem et al., 2014; Singh, 2015). The interactions are enabled through a complex network of signal molecules, including a range of volatile and non-volatile compounds, as well as *via* various metabolites (Vinale et al., 2008; Ortíz-Castro et al., 2009; Li PS et al., 2021; Nadarajah and Rahman, 2021). It is imperative that we study these microbe-plant relationships and take advantage of the intricate network of natural interactions because the two symbiotic partners jointly influence agricultural output. However, climate change and the resulting erratic weather patterns have stressed the Earth’s environment, not only in the deterioration of the soil, but also by adversely affecting crop production. Excessive use of synthetic chemicals in agriculture for increasing crop yields has led to soils that have low microbial populations making them biologically moribund. Furthermore, increasing concerns for safe environments where children visit and play has necessitated the search for eco-friendly alternatives. It is very difficult to bring a soil on life support “back to life”, but this goal is now a worldwide endeavor. Numerous studies have shown that PGPBs, a metabolically and functionally diverse group of soil bacteria, offer solutions to improve not only agricultural management, but also to restore degraded lands. Novel PGPB strains with multifunctional genetic configurations are a potent tool for restoring dead soils and helping plants cope with harsh environmental conditions. **Figure 2** demonstrates some of the well-studied phenomenon employed by beneficial root bacteria in improving the overall health of plants. Beneficial microbes help soil and crop well-being through various mechanisms, but the transition of these technologies from laboratory to field and finally to market is slow because this is a multistep process that requires the optimization of the bacterial mixture, determination of cell densities, frequency of microbial application, and selection of carriers for beneficial microorganisms. All of these require time and funding to be fine-tuned and in place for a world that is becoming hotter and drier and significantly less fertile. Because microbes behave differently in laboratory and field conditions, there is also a pressing need to propagate PGPB under field conditions for them to regain their biological activity and viability. While some beneficial bacteria have made their way into the market commercially as biostimulants, biofertilizers, biopesticides and soil amendments, many others are still in the laboratory with research being conducted on their shelf life and optimization trials. In addition to these, there are many agriculturally promising bacteria that have not made it to market for various reasons. With additional research and more information on overexpression of the desired traits of the participating strains through various -omics-based studies, a more promising environmentally based approach of eco-accommodative agriculture can be established. However, this will require a major commitment on the part of government funding agencies and commercial enterprises to bring about these goals.

## Author contributions

NK and AH compiled the research and wrote the manuscript. NK, AH and EH reviewed and edited the manuscript. EH and AJ provided the data for experiments involving *Variovorax* species. All authors contributed to the article and approved the submitted version.
