# Supplementary material for: Reviewing and renewing the use of beneficial root and soil bacteria for plant growth and sustainability in nutrient-poor, arid soils
Source: Front Plant Sci. 2023 Apr 6;14:1147535. doi: 10.3389/fpls.2023.1147535 (PMC10117987; doi:10.3389/fpls.2023.1147535)
Supplement: Supplementary file 1 [file Image_1.pdf]

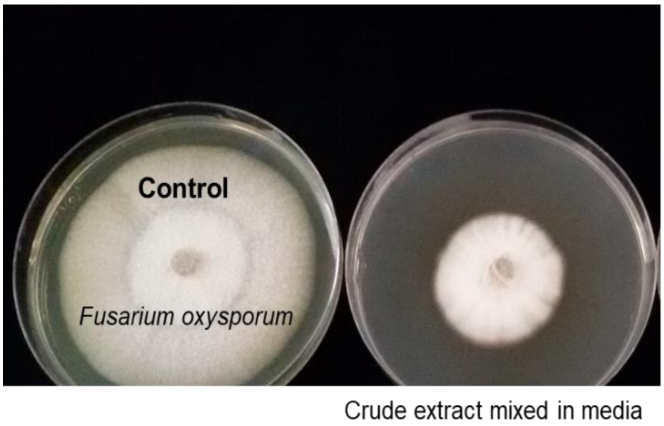

Suppl. Fig. 1. Crude BuOH (butanol) extract collected from partitioning of *B. subtilis* 30VD-1 with butanol tested against FOM: *Fusarium oxysporum* f sp. *matthioli.* A 100 µg aliquot of this dry crude extract causes 40-42% inhibition of fungal radial growth in the fungal growth bioassay.
